# Supplementary material for: Lactococcus lactis NCDO2118 exerts visceral antinociceptive properties in rat via GABA production in the gastro-intestinal tract
Source: eLife. 2022 Jun 21;11:e77100. doi: 10.7554/eLife.77100 (PMC9213000; doi:10.7554/eLife.77100)
Supplement: Supplementary file 1. [file elife-77100-supp1.docx]

|  | GAD 7 h | GAD 24 h |
| --- | --- | --- |
| NCDO2118 | 45.3 ± 4.7 | 80.2 ± 15.0 |
| NCDO2727 | 0.0 ±0.0 | 0.1 ± 0.1 |
