# Supplementary material for: Lactococcus lactis NCDO2118 exerts visceral antinociceptive properties in rat via GABA production in the gastro-intestinal tract
Source: eLife. 2022 Jun 21;11:e77100. doi: 10.7554/eLife.77100 (PMC9213000; doi:10.7554/eLife.77100)
Supplement: Supplementary file 2. — GABA production rates (µmol/min) were estimated when bacteria or control vehicle, were in presence of 0.2% (w/v) glutamate at 37°C and pH=4.4, either in 100 mM acetate buffer or with gastric content of naive rat. Two independent replicates were performed. [file elife-77100-supp2.docx]

|  | Acetate buffer | Gastric content |
| --- | --- | --- |
| NCDO2118 | 2.9 ± 1.4 | 3.0 ± 1.7 |
| NCDO2727 | Not detected | 0.8 ± 0.4 |
| Vehicle | Not detected | 0.9 ± 0.2 |
