# Supplementary material for: Lactococcus lactis NCDO2118 exerts visceral antinociceptive properties in rat via GABA production in the gastro-intestinal tract
Source: eLife. 2022 Jun 21;11:e77100. doi: 10.7554/eLife.77100 (PMC9213000; doi:10.7554/eLife.77100)
Supplement: Supplementary file 3. — Highlighted lines show conditions with significant differences in relative abundancies after Kruskal-Wallis test (p<0.07). Purple highlight indicates that the taxon is more abundant in the faecal microbiota of vehicle-treated animals, green highlight indicates that the taxon is more abundant in the faecal microbiota of NCDO2118-treated animals. [file elife-77100-supp3.docx]

| Taxa | | p-value |
| --- | --- | --- |
| 1 | g__Prevotella_Cluster_2 | 0.496291702231095 |
| 2 | g__Ligilactobacillus_Cluster_3 | 0.545349668011121 |
| 3 | g__Bacteroides_Cluster_4 | 0.545349668011121 |
| 4 | g__Lactobacillus_Cluster_5 | 0.70545698611127 |
| 5 | g__Limosilactobacillus_Cluster_6 | 0.496291702231095 |
| 6 | g__Rikenellaceae_RC9_gut_group_Cluster_7 | 0.545349668011121 |
| 7 | g__Muribaculaceae_genus_Cluster_8 | 0.0283655056052102 |
| 8 | g__Blautia_Cluster_9 | 0.289918453942572 |
| 9 | g__Lachnospiraceae_NK4A136_group_Cluster_10 | 0.256839257957856 |
| 10 | g__UCG-005_Cluster_11 | 0.289918453942572 |
| 11 | g__Odoribacter_Cluster_13 | 0.496291702231095 |
| 12 | g__Romboutsia_Cluster_17 | 0.364346126633552 |
| 13 | g__Clostridium_Cluster_22 | 0.0233422020128909 |
| 14 | g__Desulfovibrionaceae_genus_Cluster_26 | 0.130570018115735 |
| 15 | g__Turicibacter_Cluster_28 | 0.325751354478715 |
| 16 | g__Ruminococcus_gauvreauii_group_Cluster_30 | 0.762368818469847 |
| 17 | g__Oscillospiraceae_genus_Cluster_31 | 0.256839257957856 |
| 18 | g__Phascolarctobacterium_Cluster_32 | 0.545349668011121 |
| 19 | g__Paramuribaculum_Cluster_37 | 0.256839257957856 |
| 20 | g__CAG-485_Cluster_41 | 0.173617334424944 |
| 21 | g__Ruminococcus_Cluster_42 | 0.596701216729351 |
| 22 | g__Acholeplasmataceae_genus_Cluster_44 | 0.256839257957856 |
| 23 | g__Bifidobacterium_Cluster_46 | 0.173617334424944 |
| 24 | g__Alistipes_Cluster_50 | 0.496291702231095 |
| 25 | g__Akkermansia_Cluster_57 | 0.112410584655363 |
| 26 | g__Escherichia_Cluster_59 | 0.0696424047983281 |
| 27 | g__Anaerostipes_Cluster_63 | 0.130570018115735 |
| 28 | g__Eubacterium_F_Cluster_64 | 0.596701216729351 |
| 29 | g__Lachnospiraceae_genus_Cluster_74 | 0.289918453942572 |
| 30 | g__Gastranaerophilales_genus_Cluster_75 | 0.130570018115735 |
| 31 | g__UMGS1872_Cluster_77 | 0.939742989577085 |
| 32 | g__Bacteroidales_genus_Cluster_81 | 0.364346126633552 |
| 33 | g__UBA1394_Cluster_84 | 0.325751354478715 |
| 34 | g__Prevotellaceae_NK3B31_group_Cluster_85 | 0.364346126633552 |
| 35 | g__Muribaculum_Cluster_92 | 0.65014744409486 |
| 36 | g__TF01-11_Cluster_96 | 0.879829160011815 |
| 37 | g__Erysipelatoclostridium_Cluster_100 | 0.449691797968892 |
| 38 | g__Eubacterium_coprostanoligenes_group_genus_Cluster_105 | 0.762368818469847 |
| 39 | g__Schaedlerella_Cluster_107 | 0.226476066043488 |
| 40 | g__UBA3282_Cluster_115 | 0.0233422020128909 |
| 41 | g__Peptococcaceae_genus_Cluster_129 | 0.00319709944793456 |
| 42 | g__Lachnospiraceae_NK4B4_group_Cluster_134 | 0.879829160011815 |
| 43 | g__Marvinbryantia_Cluster_135 | 0.820595839755438 |
| 44 | g__UBA7160_Cluster_139 | 0.130570018115735 |
| 45 | g__Blautia_A_Cluster_140 | 0.762368818469847 |
| 46 | g__Turicimonas_Cluster_144 | 0.820595839755438 |
| 47 | g__Colidextribacter_Cluster_148 | 0.449691797968892 |
| 48 | g__ASF356_Cluster_153 | 0.173617334424944 |
| 49 | g__Eubacterium_siraeum_group_Cluster_154 | 0.150926950066717 |
| 50 | g__Eubacterium_G_Cluster_155 | 0.364346126633552 |
| 51 | g__Lachnospiraceae_NC2004_group_Cluster_159 | 0.879829160011815 |
| 52 | g__Campylobacter_Cluster_164 | 0.596701216729351 |
| 53 | g__1XD42-69_Cluster_177 | 0.879829160011815 |
| 54 | g__UBA9502_Cluster_179 | 0.545349668011121 |
| 55 | g__Enterorhabdus_Cluster_182 | 0.096303692028687 |
| 56 | g__Bacilli_genus_Cluster_185 | 0.939742989577085 |
| 57 | g__Lawsonibacter_Cluster_189 | 0.0155644113866339 |
| 58 | g__Rhodospirillales_genus_Cluster_192 | 0.70545698611127 |
| 59 | g__Defluviitaleaceae_UCG-011_Cluster_194 | 0.173617334424944 |
| 60 | g__Fusicatenibacter_Cluster_197 | 0.596701216729351 |
| 61 | g__Eubacterium_xylanophilum_group_Cluster_199 | 0.70545698611127 |
| 62 | g__Kineothrix_Cluster_203 | 0.325751354478715 |
| 63 | g__COE1_Cluster_208 | 0.545349668011121 |
| 64 | g__Phocaeicola_Cluster_209 | 0.449691797968892 |
| 65 | g__Oscillibacter_Cluster_214 | 0.256839257957856 |
| 66 | g__Lachnospiraceae_UCG-006_Cluster_216 | 0.879829160011815 |
| 67 | g__Eubacterium_fissicatena_group_Cluster_218 | 0.226476066043488 |
| 68 | g__Ruminococcus_torques_group_Cluster_226 | 0.545349668011121 |
| 69 | g__Eubacterium_Cluster_229 | 0.325751354478715 |
| 70 | g__CAG-95_Cluster_234 | 0.596701216729351 |
| 71 | g__Roseburia_Cluster_248 | 0.879829160011815 |
| 72 | g__UMGS1994_Cluster_255 | 0.70545698611127 |
| 73 | g__UCG-003_Cluster_258 | 0.289918453942572 |
| 74 | g__Frisingicoccus_Cluster_269 | 0.0412500165939395 |
| 75 | g__Duncaniella_Cluster_278 | 0.820595839755438 |
| 76 | g__Emergencia_Cluster_280 | 0.939742989577085 |
| 77 | g__Erysipelotrichaceae_UCG-003_Cluster_285 | 0.173617334424944 |
| 78 | g__AM07-15_Cluster_296 | 0.65014744409486 |
| 79 | g__Papillibacter_Cluster_308 | 0.0412500165939395 |
| 80 | g__Rothia_Cluster_313 | 0.70545698611127 |
| 81 | g__Streptococcus_Cluster_312 | 0.596701216729351 |
| 82 | g__Acutalibacter_Cluster_320 | 0.65014744409486 |
| 83 | g__Lachnospiraceae_UCG-001_Cluster_323 | 0.496291702231095 |
| 84 | g__Lachnospiraceae_FCS020_group_Cluster_336 | 0.112410584655363 |
| 85 | g__Lachnoclostridium_Cluster_339 | 0.65014744409486 |
| 86 | g__Anaerotruncus_Cluster_341 | 0.545349668011121 |
| 87 | g__Murimonas_Cluster_346 | 0.939742989577085 |
| 88 | g__NK4A214_group_Cluster_353 | 0.496291702231095 |
| 89 | g__A2_Cluster_361 | 0.65014744409486 |
| 90 | g__Clostridia_vadinBB60_group_genus_Cluster_363 | 1 |
| 91 | g__UMGS1815_Cluster_369 | 0.762368818469847 |
| 92 | g__Anaerosacchariphilus_Cluster_373 | 0.325751354478715 |
| 93 | g__14-2_Cluster_429 | 0.289918453942572 |
| 94 | g__Clostridia_UCG-014_genus_Cluster_435 | 0.226476066043488 |
| 95 | g__Paludicola_Cluster_440 | 0.939742989577085 |
| 96 | g__Ruthenibacterium_Cluster_460 | 0.112410584655363 |
| 97 | g__Christensenellaceae_R-7_group_Cluster_481 | 0.173617334424944 |
| 98 | g__Tuzzerella_Cluster_486 | 0.112410584655363 |
| 99 | g__Eubacterium_brachy_group_Cluster_493 | 0.496291702231095 |
| 100 | g__Family_XIII_UCG-001_Cluster_510 | 0.00319709944793456 |
| 101 | g__Parabacteroides_Cluster_509 | 0.762368818469847 |
| 102 | g__OEMS01_Cluster_516 | 0.939742989577085 |
| 103 | g__Enterococcus_Cluster_527 | 0.226476066043488 |
| 104 | g__Ruminococcaceae_genus_Cluster_539 | 0.096303692028687 |
| 105 | g__CAG-41_Cluster_540 | 0.65014744409486 |
| 106 | g__GCA-900066575_Cluster_548 | 0.325751354478715 |
| 107 | g__Eubacterium_nodatum_group_Cluster_546 | 0.939742989577085 |
| 108 | g__Butyricicoccus_Cluster_565 | 0.289918453942572 |
| 109 | g__Candidatus_Stoquefichus_Cluster_619 | 0.879829160011815 |
| 110 | g__Lachnospirales_genus_Cluster_670 | 0.150926950066717 |
| 111 | g__Clostridia_genus_Cluster_693 | 0.596701216729351 |
| 112 | g__Prevotellamassilia_Cluster_699 | 0.939742989577085 |
| 113 | g__Eisenbergiella_Cluster_707 | 0.820595839755438 |
| 114 | g__Stoquefichus_Cluster_717 | 0.364346126633552 |
| 115 | g__Peptococcus_Cluster_722 | 0.496291702231095 |
| 116 | g__CAG-56_Cluster_749 | 0.545349668011121 |
| 117 | g__Candidatus_Soleaferrea_Cluster_747 | 1 |
| 118 | g__Eubacterium_J_Cluster_775 | 0.325751354478715 |
| 119 | g__Adlercreutzia_Cluster_789 | 1 |
| 120 | g__CAG-508_genus_Cluster_799 | 0.939742989577085 |
| 121 | g__Prevotellaceae_UCG-001_Cluster_803 | 0.879829160011815 |
| 122 | g__Bacteria_genus_Cluster_817 | 0.545349668011121 |
| 123 | g__Candidatus_Saccharimonas_Cluster_840 | 0.939742989577085 |
| 124 | g__CAG-110_Cluster_871 | 0.325751354478715 |
| 125 | g__Coprococcus_Cluster_875 | 0.879829160011815 |
| 126 | g__Flavonifractor_Cluster_893 | 0.130570018115735 |
| 127 | g__Staphylococcus_Cluster_902 | 0.496291702231095 |
| 128 | g__Family_XIII_AD3011_group_Cluster_948 | 0.596701216729351 |
| 129 | g__QXXE01_Cluster_954 | 0.405678895285055 |
| 130 | g__Christensenellaceae_genus_Cluster_978 | 0.150926950066717 |
| 131 | g__CAG-81_Cluster_999 | 0.496291702231095 |
| 132 | g__MD308_Cluster_1054 | 0.596701216729351 |
| 133 | g__Anaerovorax_Cluster_1050 | 0.449691797968892 |
| 134 | g__An92_Cluster_1053 | 0.939742989577085 |
| 135 | g__Parasutterella_Cluster_1044 | 0.820595839755438 |
| 136 | g__Dysosmobacter_Cluster_1099 | 0.173617334424944 |
| 137 | g__Oscillospirales_genus_Cluster_1076 | 0.545349668011121 |
| 138 | g__D16-34_Cluster_1121 | 0.405678895285055 |
| 139 | g__Eubacterium_hallii_group_Cluster_1206 | 0.879829160011815 |
| 140 | g__Angelakisella_Cluster_1290 | 0.545349668011121 |
| 141 | g__Erysipelatoclostridiaceae_genus_Cluster_1268 | 0.130570018115735 |
| 142 | g__Desulfovibrio_Cluster_1285 | 0.939742989577085 |
| 143 | g__Mediterraneibacter_Cluster_1304 | 0.198764606373234 |
| 144 | g__UCG-010_genus_Cluster_1319 | 0.150926950066717 |
| 145 | g__Bilophila_Cluster_1307 | 0.70545698611127 |
| 146 | g__Acetatifactor_Cluster_1361 | 0.130570018115735 |
| 147 | g__Intestinimonas_Cluster_1355 | 0.364346126633552 |
| 148 | g__Defluviitaleaceae_genus_Cluster_1422 | 0.939742989577085 |
| 149 | g__Allobaculum_Cluster_1858 | 0.762368818469847 |
