# Supplementary material for: Lactococcus lactis NCDO2118 exerts visceral antinociceptive properties in rat via GABA production in the gastro-intestinal tract
Source: eLife. 2022 Jun 21;11:e77100. doi: 10.7554/eLife.77100 (PMC9213000; doi:10.7554/eLife.77100)
Supplement: Supplementary file 4. — Primers used for the inactivation of gadB in Lactococcus lactis NCDO2118. [file elife-77100-supp4.docx]

| Primer name | *5’-3’ sequence* |  |
| --- | --- | --- |
| 821-GBgadCR | ggaattcgatTTAGATGCCATAGGAGGATTTTC | Amplification of *gadB* upstream sequence |
| 894-GBgadC2F | GATGAATATCGTACATCCTCCAATTTTTTAATG |  |
| 892-GBkefA2F | AAGCTTGATAAAACAAGAAAATATTCATGAAATTCAG | Amplification of *gadB* downstream sequence |
| 893-GBkefA2R | GGAGGATGTACGATATTCATCTTAAGAAAAATCAAAAGC |  |
| 822-GBpGhost9EVF | catctaaATCGAATTCCTGCAGCCCG | Amplification of pGhost9 backbone |
| 891-GBpGh9EV2R | TCTTGTTTTATCAAGCTTATCGATACCGTC |  |
| 898-amt GadB(DCO) | TTGGATTAGCTGCGGCATATTTTATCG | Verification of *gadB* deletion onto the chromosome |
| 899-avl GadB(DCO) | CCTTGTTGACCATAATGCAAAGCAGGT |  |

| Primer name | *5’-3’ sequence* |
| --- | --- |
| GabSeq_2_F | AAAATATAGAAGGAGACTATTGCAAATAGC |
| GabSeq_2_R | AAAAATTAATGGCCATCGTTGGTAGTTCTC |
| GabSeq_3_F | TCTGTGCAGCAGAAATGGCGACGGTTGAA |
| GabSeq_3_R | TCCCCATAAATTTTTCTTTTTCACTCGCAT |
| GabSeq_4_F | CGGTTATTCCTCAAAAAGACTTATCATTAA |
