# Supplementary material for: Lactococcus lactis NCDO2118 exerts visceral antinociceptive properties in rat via GABA production in the gastro-intestinal tract
Source: eLife. 2022 Jun 21;11:e77100. doi: 10.7554/eLife.77100 (PMC9213000; doi:10.7554/eLife.77100)
Supplement: Transparent reporting form [file elife-77100-transrepform1.docx]

***eLife’s* transparent reporting form**

We encourage authors to provide detailed information *within their submission* to facilitate the interpretation and replication of experiments. Authors can upload supporting documentation to indicate the use of appropriate reporting guidelines for health-related research (see [EQUATOR Network](http://www.equator-network.org/%20)), life science research (see the [BioSharing Information Resource](https://biosharing.org/)), or the [ARRIVE guidelines](http://www.plosbiology.org/article/info:doi/10.1371/journal.pbio.1000412) for reporting work involving animal research. Where applicable, authors should refer to any relevant reporting standards documents in this form.

If you have any questions, please consult our Journal Policies and/or contact us: [editorial@elifesciences.org](mailto:editorial@elifesciences.org).

**Sample-size estimation**

- You should state whether an appropriate sample size was computed when the study was being designed
- You should state the statistical method of sample size computation and any required assumptions
- If no explicit power analysis was used, you should describe how you decided what sample (replicate) size (number) to use

Please outline where this information can be found within the submission (e.g., sections or figure legends), or explain why this information doesn’t apply to your submission:

**Work involving animal research for visceral sensitivity measurement**

Ethical statement: Animals were used by strictly following the national and European guidelines and laws regarding the use of animals in experimentation (EU Directive 2010/63/EU) and according to the ARRIVE guidelines. All experiments were approved by the Local Animal Care and Use Committee (APAFIS#5577-201606061639777v3 and APAFiS#14898-2018043016031426).

Study design, experimental animals, housing, experimental procedures: see sections Materials and Methods, Animals and surgical procedure (4.3), Colorectal distension procedure and acute stress procedure (4.4), Experimental protocol for *in vivo* assays under PRS conditions (4.5.1).

Sample size: no power calculation was used. In this preclinical study, for each set of experiments, groups of 7 to 12 female rats were considered by performing 6 independent series with animals representative of each group (2 animals/group). The variable number of animals per group is explained by the fact that during the course of the 10-day oral treatment, some animals lost their equipment for electromyography (EMG) recording.

**Replicates**

- You should report how often each experiment was performed
- You should include a definition of biological versus technical replication
- The data obtained should be provided and sufficient information should be provided to indicate the number of independent biological and/or technical replicates
- If you encountered any outliers, you should describe how these were handled
- Criteria for exclusion/inclusion of data should be clearly stated
- High-throughput sequence data should be uploaded before submission, with a private link for reviewers provided (these are available from both GEO and ArrayExpress)

Please outline where this information can be found within the submission (e.g., sections or figure legends), or explain why this information doesn’t apply to your submission:

**Culture of bacterial strains:** bacterial cultures in bioreactor for each strain under study were performed in duplicate (i.e. independent biological replicates). See section Materials and Methods Bacterial strains, medium and culture conditions (4.1).

**Measurement of GABA concentration all along the gastro-intestinal tract:** the number of animals considered for this study is 5 (see section Materials and Methods Experimental protocol for *in vivo* assays without PRS (4.5.2) and GABA extraction and quantification from 200 mg of contents (4.7).

**Measurement of specific GAD activity:** for each of the two cultures independent biological replicates in bioreactor, three technical replicates (n=3) were considered. See legend of Table S1.

***In vitro* kinetics of GABA production:** Two independent biological replicates were performed. See legend of Supplementary File 2 (Table).

**Statistical reporting**

- Statistical analysis methods should be described and justified
- Raw data should be presented in figures whenever informative to do so (typically when N per group is less than 10)
- For each experiment, you should identify the statistical tests used, exact values of N, definitions of center, methods of multiple test correction, and dispersion and precision measures (e.g., mean, median, SD, SEM, confidence intervals; and, for the major substantive results, a measure of effect size (e.g., Pearson's r, Cohen's d)
- Report exact p-values wherever possible alongside the summary statistics and 95% confidence intervals. These should be reported for all key questions and not only when the p-value is less than 0.05.

Please outline where this information can be found within the submission (e.g., sections or figure legends), or explain why this information doesn’t apply to your submission:

A specific section “Statistical methods” is included at the end of the Materials and Methods part (4.9), with a particular focus on animal experiments. Data are reported as the means ± standard errors of the means (SEM). The software GraphPad Prism 8.3 (GraphPad, San Diego, CA) was used for statistical analysis. For visceral sensitivity evaluation, one-way ANOVA, followed by Tukey’s Multiple Comparison test, was performed to compare data between the different groups of animals. Statistical significance was accepted at P < 0.05. P-values vs. reference groups (*P<*0.05; *P<*0.01; *P<*0.001; P<0.0001) are indicated in the legend of Figures 2 and 3.

For GABA measurements in the gastro-intestinal tract, data are reported as the means ± SEM and the non-parametric Kruskal-Wallis test, supplemented by Dunn's multiple comparison test, was used. Statistical significance was accepted at P < 0.05.

Data analysis for faecal microbiota composition using 16S rRNA gene sequencing is provided separately (4.8) including bio-informatic and statistical treatments.

(For large datasets, or papers with a very large number of statistical tests, you may upload a single table file with tests, Ns, etc., with reference to sections in the manuscript.)

**Group allocation**

- Indicate how samples were allocated into experimental groups (in the case of clinical studies, please specify allocation to treatment method); if randomization was used, please also state if restricted randomization was applied
- Indicate if masking was used during group allocation, data collection and/or data analysis

Please outline where this information can be found within the submission (e.g., sections or figure legends), or explain why this information doesn’t apply to your submission:

Randomization was used for allocating animals into the different experimental groups. Randomization was also used for visceral sensitivity measurements between animals from each group. No masking was used for data collection and analysis. This information is not given in the current version of the manuscript but could be added if necessary.

**Additional data files (“source data”)**

- We encourage you to upload relevant additional data files, such as numerical data that are represented as a graph in a figure, or as a summary table
- Where provided, these should be in the most useful format, and they can be uploaded as “Source data” files linked to a main figure or table
- Include model definition files including the full list of parameters used
- Include code used for data analysis (e.g., R, MatLab)
- Avoid stating that data files are “available upon request”

Please indicate the figures or tables for which source data files have been provided:

Relevant additional data files are provided as source data files.

For figures 1A 1B and S3: Excel file.

For figures 2A 2B, 3A 3B, 4, S1 and S2: txt format.

For figure 5: All data (raw and treated) can be found in this link: <https://forgemia.inra.fr/umrf/exploremetabar>
